# Gap Junction–Mediated Communication in Melanoma: From Tumor Progression to Treatment Response

**DOI:** 10.3390/ijms27062705

**Published:** 2026-03-16

**Authors:** Juliana Massoud, Sarah Ibrahim, Madison Jensen, Michael C. Beary, Ben Nafchi, Michael Springer, Shoshanna N. Zucker

**Affiliations:** D’Youville University School of Pharmacy, D’Youville University, 320 Porter Avenue, Buffalo, NY 14201, USA; massoj15@dyc.edu (J.M.); ibras09@dyc.edu (S.I.); jensem29@dyc.edu (M.J.); bearym23@dyc.edu (M.C.B.); alimob19@dyc.edu (B.N.); sprinm22@dyc.edu (M.S.)

**Keywords:** melanoma, gap junctions, connexins, tumor progression

## Abstract

Melanoma is a highly malignant neoplasm of the skin with early metastatic spread and increasing incidence worldwide. Although there are significant therapeutic advances in immunotherapy, especially with the checkpoint inhibitors targeting PD-1 and CTLA-4, challenges such as treatment-related toxicities, a heterogeneous response to therapy, and drug resistance continue to exist. There are unmet needs for novel therapeutic strategies and/or approaches to complement the existing treatment options. Potential targets for future melanoma treatment are the gap junction proteins, connexins, which show an altered pattern of regulation during melanoma progression. In this review, we highlight the regulation of gap junctions during melanoma progression and the characterization of gap junctions as tumor suppressors during early-stage tumor development and then the reversion to enhancers of tumor metastasis during late-stage melanoma progression. We provide a comprehensive overview of gap junctions in the skin and how the connexin proteins, which comprise gap junctions, are alternatively regulated in melanoma progression. Connexins are protein channels in the human body that consist of 21 isoforms. These isoforms form gap junctions that provide important intercellular signaling and permeability channels. Each connexin protein consists of four transmembrane domains and a C-terminal tail, which is an important part of its function and regulation. Permeants of gap junctions include signaling molecules such as cyclic AMP and inositol triphosphate which are linked to key cellular behaviors such as proliferation and migration, making them essential for several tumor-related processes. At least ten connexin isoforms are found in normal skin. Connexin 43 (Cx43) is classified as the most prevalent isoform while Connexin 26 (Cx26) has been reported to be more specialized with restricted expression patterns. Cx43 and Cx26 regulate the growth, differentiation, and repair of the epidermis after injury. Evidence suggests that connexins have a stage-related function in melanoma. Loss of connexin expression and gap junctional intercellular communication is linked to tumor suppression and loss of differentiation in early-stage melanoma, while re-expression or overexpression of specific connexins, notably Cx43, may promote metastasis through enhanced tumor–stromal interactions and increased motility in late-stage melanoma. Such opposing actions of connexins support their candidacy as biomarkers and therapeutic targets. Understanding the dual-stage related functions of connexins in melanoma development and progression may lead to less cytotoxic and more efficient future therapeutic approaches.

## 1. Melanoma Progression—Limitations of Current Therapies

### 1.1. Epidemiology and Public Health Burden of Melanoma

Melanoma represents a clinically significant form of skin cancer due to both its invasive characteristics and increasing incidence worldwide. Although melanoma only represents less than 5% of the total incidence of skin cancer, it is much more dangerous in terms of morbidity and mortality, especially in the advanced stages of the disease [1]. Public health relevance is reflected in the treatment burden on the health system in terms of expenditure and resources consumed, particularly in high-risk countries such as Australia and New Zealand [2]. The management of melanoma is a considerable burden on the public, further emphasizing its importance from both a clinical and economic perspective. With the growing awareness of the impact of melanoma continuing to evolve, the pursuit of further studies must focus on improving treatments for the disease, particularly since the continued burden continues to weigh heavily on the public health system internationally [3].

### 1.2. Genetic Drivers and Environmental Risk Factors

Furthermore, melanoma represents the result of multiple genetic mutations and environmental risk factors. Several driver mutations have been identified in melanocytic neoplasms characterized by dysregulated cell proliferation, the most common ones being BRAF and NRAS [2]. Meanwhile, environmental factors, such as extended and discontinuous ultraviolet exposure, create DNA damage, thus increasing further mutation in genetically predisposed patients and promoting the development of this skin cancer [4]. These mutations represent aggressiveness and tumorigenicity in melanoma. Moreover, a considerable percentage of early-stage melanoma cases at stage I and II also progress to metastatic melanoma [5], highlighting their contribution to early dissemination and metastasis. Such molecular factors indicate the necessity for an innovative approach regarding prevention and treatment to establish personalized medicine in the field of melanoma therapy.

### 1.3. Melanoma Staging and Prognostic Determinants

A precise understanding of the staging of melanoma is also necessary to determine the prognosis and therapeutic management of each patient. The major classification system for melanoma is the American Joint Committee on Cancer 8th edition TNM system, which classifies the disease from the earliest localized in situ tumors (stage 0) to the advanced metastatic cases (stage IV), by such variables as presence of ulceration, thickness, and the degree of involvement of the regional lymph nodes [6]. Early diagnoses or early stages of melanomas confined to the epidermis or with a thin dermal invasion below 1 mm may have a far better prognosis. On the other hand, distant spread and involvement of regional lymph nodes are considered significant prognostic factors associated with the lowest survival rate [7]. Melanoma may be managed according to various therapeutic options, given the stage: while localized tumors require only surgical excision, as with the more advanced stages, therapeutic interventions become more complex: sentinel lymph node biopsy, systemic therapies, and protracted follow-up. Detection of the early stage affects not only the prognosis but also the therapeutic strategies and schedules. Thus, accurate clinical staging and subsequent development of therapeutic decisions may help the clinician in view of predicting the most probable clinical course of the disease in patients with melanoma [8].

### 1.4. Advances in Immunotherapy and Emerging Therapeutic Strategies

More melanoma treatment trends in recent studies show that they have been focused on immunotherapeutic approaches, with leading participation of immune checkpoint inhibitors in modern schemes of treatment. Immune checkpoint inhibitors target mostly cellular pathways such as PD-1/PD-L1 and CTLA-4, releasing the inhibitory signals on cytotoxic T lymphocytes and restoring the classical anti-tumor immune response. Also, the progress and regulatory approval of TIL transfer and CAR T-cell therapy have yielded clinical progress to advanced or refractory melanoma patients, thus expanding the frame of immunotherapeutic possibilities [9]. For example, recent advances signal the possible interaction of immunotherapy with other therapeutic technologies, targeting epigenetic drugs. In these treatments, targeting epigenetic regulators might reduce resistance while successively potentiating the immune effect clearance of the tumor [10]. Introduction of these therapies into clinical practice has managed to expand melanoma treatment options and guarantees further hope in breaking conventional treatment boundaries.

### 1.5. Limitations of Current Therapies and Mechanisms of Resistance

Although immunotherapy has revolutionized the treatment of melanoma by expanding overall survival for a significant number of patients, major limitations remain. Immunotherapy can cause adverse effects systems, which can be attributed to the fact that most of these therapies do not have sufficient selectivity and thus target normal, healthy issues in addition to tumor cells. In addition, the emergence of resistance remains a significant challenge, characterized by the capability of tumor cells to adapt to therapy and reduce its efficacy over time, through genetic or epigenetic alterations [11]. These resistance mechanisms can entail modifications in the cell signaling pathways involved in the immune targeting of neoplasia, the tumoral microenvironment, or mutations that allow evasion of immune recognition and cytotoxic mechanisms. Naming such challenges, understanding resistance and adverse effects as a multifactorial process continues to provide insights for alternatives, including therapies with epigenetic regulators, with the goal of overcoming resistance and improving selectivity [12].

### 1.6. Rationale for Novel and Targeted Therapeutic Approaches

In this context, the recurring challenges associated with contemporary treatment options for melanoma suggest that prioritization of alternative treatment strategies that meet both the efficacy and safety for the patients’ standards is required. Given the considerable level of immune-related adverse events and tumor adaptation by means of resistance mechanisms, there is still an evident demand for strategies with even more pronounced specificity and reduced off-target impact. Implementation of novel strategies, which are based on molecular rearrangements that are unique for melanoma cells, are likely to be successful in the future. A specific example is the epigenetically deregulated processes, which are believed to establish a new trend in the development of approaches that can cause significantly less collateral damage for healthy tissues and simultaneously reduce disease advancement [13]. Targeting epigenetic regulators may disrupt the tumor sustainability networks with much higher precision in a way to provide overcoming resistance and enhance the long-term effectiveness of melanoma therapy. Therefore, the existing rationale for expanding preclinical and clinical investigation in this area is aimed at detecting interventions that will ensure both durability and tolerability improvement of the melanoma therapy [14]. Due to the altered regulation of gap junctions during melanoma progression, they represent a novel target for therapeutic regulation.

## 2. Gap Junctions—Structure and Function

### 2.1. History of Gap Junctions

In 1966, research conducted by Loewenstein and Kanno using electrophysiological techniques was pivotal in advancing the understanding of gap junctions as pathways for direct cell-to-cell communication [15]. The ability for cells to communicate signals to one another was a hypothesized theory that Lowenstein and Kanno were able to directly support via electrophysiological techniques. Key observations of the 1966 study include the discovery of electrical coupling, uncoupling in cancer cells, the calcium hypothesis, and cell growth regulation. All contributed to the understanding of the several molecular pathways and certain proteins cells used to communicate directly [15].

Electrical coupling is the process of the direct flow of ions between adjacent cells and is implicated in processes including heart muscle contraction and action potentials. Loewenstein’s lab reported a lack of ionic coupling in hepatoma (liver cancer) cells compared to normal liver cells. This led to the foundational hypothesis that a loss of gap junctional intercellular communication (GJIC) is a general phenotypic aspect of cancer cells and may be involved in the loss of growth control associated with tumors [15]. Lowenstein and Kanno also hypothesized that gap junctions help regulate overall tissue growth. They observed that in normal, non-cancerous liver cells, adjacent cells are chemically and electrically coupled so that small ions and cells can pass from the cytoplasm of one cell to another, which plays a critical role in controlling when cells grow, divide, differentiate or stop proliferating [15]. When they observed the cancerous liver cells (hepatoma), they found an absence or severe reduction in this coupling- cancer cells lacked functional gap-junction communication. From this, they hypothesized that loss of intercellular communication removes “growth control” and thereby allows cells to proliferate unchecked- the hallmark of cancerous tissues [15].

More recent research explores the critical roles of connexins and gap junctions in the progression of cancer. Gap junctions, formed by connexin proteins, are essential for cell-to-cell communication and play a key role in regulating cell growth, differentiation, and tissue homeostasis [16]. Reduced expression of connexins, especially Cx43, is commonly observed in various cancers, including breast, lung, and liver cancers. This loss of gap junction communication results in disrupted cell signaling, which allows tumor cells to grow uncontrollably [16]. Studies also suggest potential for connexins to influence tumor microenvironments, suggesting that changes in gap junctions can affect not only cancer cell behavior but also the surrounding stromal cells, which support tumor growth and metastasis, emphasizing the dual roles of connexins in cancer [16]. While some connexins, such as Cx26 and Cx32, may act as tumor suppressors, their loss can promote cancer progression. On the other hand, some evidence suggests that connexins like Cx43 may have both pro-tumorigenic and anti-tumorigenic effects depending on the context, such as the stage of cancer or the specific cellular environment [16]. Targeting connexins and their associated gap junctions offers potential therapeutic strategies to regulate cell communication and possibly restore normal cell behavior in cancerous tissues [16].

Recent research has improved our understanding of how connexin channels and hemichannels work, how they are linked to disease, and how they interact with other proteins [17]. Although it is clear that connexins influence many cellular processes in nearly every organ, an important challenge remains: determining how specific connexins function in different tissues. This is complicated by limited knowledge of which molecules pass through each type of channel in living tissues and by the fact that different connexins can combine to form mixed channels [17]. As more connexins are linked to disease, researchers from many medical and biological fields are becoming interested in gap junctions as potential therapeutic targets. This growing interest is expected to greatly improve our understanding of connexin-based communication throughout the body and their effects on cancer progression [17].

### 2.2. Classification and Structure

Gap junctions are specialized intercellular channels that enable direct communication between adjacent cells by allowing ions, metabolites, and small signaling molecules (typically <1.5 kDa) to pass from the cytoplasm of one cell to the cytoplasm of the adjacent cell [1]. These channels are formed by connexins, four-pass transmembrane proteins, which oligomerize into hexamers called connexons. When a connexon on one cell comes into physical contact with a connexon on a neighboring cell, they create a continuous aqueous pore [1]. This pore essentially becomes a channel where cells may communicate with ease. Therefore, gap junctions play essential roles in maintaining tissue homeostasis, synchronizing cellular activities, and coordinating rapid physiological responses, such as electrical conduction in cardiac muscle and metabolic coupling (the process of linking exergonic and endergonic reactions) in epithelial tissues [1]. Connexins also have non-channel roles, including cell adhesion and gene expression. There is significant research that also suggests that gap junctions play a role in cancerous tissue growth [18,19]. Overall, gap junctions enable cells to efficiently and effectively communicate through both intercellular channels and signal transduction pathways to promote tissue homeostasis.

Connexin composition varies across tissues, and different connexin isoforms confer unique permeability and gating properties [20]. Gap junction channels are dynamically regulated by pH, calcium levels, phosphorylation, and cellular stress, allowing cells to modulate communication according to physiological needs. Disruption of gap junction signaling, either through connexin mutations or dysregulation, is associated with a wide range of diseases, including cardiac arrhythmias, peripheral neuropathies, and certain skin disorders including cancer, i.e., melanoma [20]. Research in molecular biology continues to uncover how connexins interact with intracellular proteins and signaling pathways, highlighting their importance beyond channel formation, expanding into broader roles in gene regulation [20].

In humans, there are 21 connexin family members. They are named either by their molecular weight (e.g., Cx43) or by their gene nomenclature (e.g., GJA1). Overall, there are five classes, or subfamilies, of connexins- alpha, beta, gamma, delta, and epsilon which are classified based on sequence similarity and the potential to form heteromeric gap junctions with adjacent cells. The classifications of gap junctions are outlined in Table 1. The potential for gap junctions to form heteromeric channels is limited to the specific subtype of the connexin protein as highlighted below [21].

The molecular weight of each connexin differs based primarily on the length of their C-terminal tail. The C-terminal tail is an important structure of the connexin because it regulates channel gating (opening and closing) and has multiple binding and phosphorylation domains [21]. Phosphorylation of the C-terminal tail (by kinases such as PKC, MAPK, Src, CK1, and PKA) can change channel open probability, trafficking, assembly, degradation, and even the switch between forming functional gap junctions versus undocked hemichannels [22,23].

### 2.3. Intercellular Communication and Activating Pathways

Intercellular communication via gap junctions is an essential mechanism by which adjacent cells coordinate physiological and biochemical activities to maintain tissue homeostasis—a stable and balanced cellular environment. Gap junctions are specialized membrane structures composed of connexin proteins that assemble into hexameric hemichannels, or connexons, which align between neighboring cells to form aqueous pores. These channels permit the direct transfer of ions, metabolites, and small signaling molecules—such as calcium, cyclic nucleotides, and inositol trisphosphate, etc. They therefore enable rapid, contact-dependent communication that bypasses extracellular signaling pathways. Through this direct exchange, gap junctions play a critical role in synchronizing cellular responses during processes including development, differentiation, electrical conduction, and stress signaling [21].

Intercellular communication, the process by which cells send and receive signals from one another, affects tissue growth, particularly in the context of cancer. A core finding of the gap junctions is that, in cancerous tissues, gap junction communication is often impaired or absent [15]. This loss of cellular coordination leads to uncontrolled cell proliferation, a hallmark of cancer. When normal intercellular signaling pathways are disrupted, cancer cells are no longer responsive to growth-regulatory signals, such as those that would normally limit cell division or induce cell death [15]. This lack of communication between cells in a tissue allows cancer cells to grow without the normal checks and balances that would occur in healthy tissues. There is also experimental evidence showing that normal cells, when introduced into cancerous environments, can restore some degree of gap junction communication, leading to a more controlled growth pattern. This observation supports the idea that restoring proper cellular communication could potentially halt or slow cancer progression [15].

Intracellular signaling pathways mediated through gap junctions are a critical mechanism by which signaling events are coordinated across cells. Gap junctional communication enables intracellular signaling networks at the tissue level, ensuring coordinated responses to physiological stimuli and contributing to processes such as development, metabolic regulation, and maintenance of cellular homeostasis. All of these processes are essential to maintain healthy, living cells throughout the human body [23]. Gap junctions are found in many tissue types, including epithelial, cardiac, and neuronal tissues, where they are essential for maintaining tissue homeostasis and function [24]. For example, in the heart, gap junctions enable the synchronized contraction of cardiac muscle by allowing electrical signals to spread rapidly between cells [1]. The function of gap junctions is tightly regulated, and their opening or closing can be influenced by various factors, including cellular signaling pathways, changes in pH, and calcium ion concentration. The permeability of gap junctions can vary depending on the connexin subtype that forms the channel. Different connexins, such as Cx43 in the heart and Cx26 in the cochlea, can form gap junctions with distinct properties, allowing for tissue-specific regulation of intercellular communication [24]. Gap junctions are also dynamic structures, as they can be regulated by phosphorylation or degradation in response to cellular signaling events, allowing cells to modulate communication in response to changing environmental conditions or during development [25].

## 3. Gap Junctions in Normal Skin & Melanoma

### 3.1. Structure and Function of Gap Junctions in Normal Skin

The unique architecture of gap junctions is fundamental to tissue homeostasis as it allows for coordinated responses in processes such as cell proliferation, differentiation, and metabolic stability [26]. In the human skin, gap junctions contribute to the coordinated mechanisms that mediate tissue homeostasis such as wound healing, barrier function, and growth. By controlling the flow of signaling molecules and metabolites between connected neighbors, gap junctions play an essential role in supporting vital processes of the skin [26]. Moreover, human epidermis contains numerous different connexin isoforms, each fulfilling their own unique epidermal tasks. This fact is indicative of the stratified and dynamic nature of connexin expression in layers of epithelium, where specific proteins are homed to basal, spinous or granular parts of the epidermis, respectively, to preserve the architecture and communication of the cells formed [27]. 

### 3.2. Connexin Diversity in Human Skin: Isoforms and Epidermal Localization

In total, 21 identified connexin isoforms are products of the five gap junction gene families in the human body: GJA, GJB, GJC, GJD, and GJE. These five gene families give rise to Cx23, Cx26, Cx30, Cx30.3, Cx31, Cx31.1, Cx32, Cx36, Cx37, Cx40, Cx43, Cx45, Cx46, Cx50, Cx57, and the rarely mentioned neuronal and tissue-specific variants Cx31.9, Cx34.1 along with other connexins from the α, β, γ, δ, and ε subfamilies [28]. The molecular weights, permeability, gating characteristics, and tissue-localization are all different for each isoform. In total, these 21 connexins represent the complete set of human proteins that can form gap junctions. Of the 21 recognized connexin isoforms in the human body, at least 10 are expressed in skin, with the six major epidermal connexins including Cx26, Cx30, Cx30.3, Cx31, Cx31.1 and Cx43 predominantly localized to keratinocytes and the remaining, Cx37, Cx40, Cx45 and Cx46, detected in dermal vasculature, smooth muscle, adnexal structures and hair follicles. The connexins expressed in the epidermis are outlined in Table 2. Among these isoforms, this review focuses mainly on connexin 26 and connexin 43 due to their unique expression patterns and roles in normal and disrupted conditions. By forming intercellular channels (gap junctions), connexins allow trafficking of metabolites, ions, and signaling molecules that are obligatory for the coordinated response of the tissue [29]. Unique patterns of expression and isoforms functionality constitute the homeostatic control network of the epidermis and its dysregulation, when altered, points to the dispersive nature of skin tissue.

### 3.3. Comparative Roles of Major Epidermal Connexins: Focus on Cx26 and Cx43

Connexin 43 (Cx43) is the most widely distributed isoform found in the skin and facilitates the expression of intercellular communication networks that support epidermal cellular process, including tissue proliferation, differentiation and metabolic activity across the layers of the epidermis. The widespread expression of Cx43 is consistent with its physiological role in the epidermis and provides a critical platform for intercellular connectivity through the formation of gap junction channels [27]. The generation of intercellular junctions by wild-type Cx43 occurs without formation of constitutively active hemichannels in normal physiological conditions. The incorporation of mutants of Cx43 into the hemichannels may promote varying functional activity and provide insight into dysregulation of this protein and its contributions to skin disease and abnormal epidermal architecture [27].

On the other hand, Cx26 is more locally expressed in the skin in defined regions of the epidermis and mucosal epithelia. This allows for Cx26 to participate in more selective intercellular communication networks and to achieve unique physiological and regulatory functions critical to tissue compartmentalization. Cx26 assembles gap junction channels that allow the passage of ions and signaling molecules, and deregulation of Cx26 activity has been linked to epithelial diseases, including inflammatory and inherited skin disorders [29]. Both Cx26 and Cx43 have an important impact on the coordination of proinflammatory signaling through ATP efflux during immune activation. Overall results reveal the unique, localized roles of Cx26 in protecting epithelial integrity and mediating specifically regulated signaling between skin cells.

### 3.4. Functional Implications in Growth, Differentiation, and Epidermal Homeostasis

The physiological roles of gap junctions and their connexin subtypes are also essential for skin development control by regulating keratinocyte proliferation and differentiation. The inter-keratinocyte communications via gap junctions mainly composed by Cx43 are responsible for the permeable intercellular passages that enable the distribution of ions and small secondary messengers responsible for synchronized keratinocyte cell cycle proliferation and differentiation [30]. Functional studies show that Cx43 knockdown abolishes gap junctional communication and leads to growth impairment along with reduced skin-derived precursor cell expansion. In addition to acting as gap-junction forming channels, Cx43 has been reported to regulate the metabolic machinery and confine the cellular organization in gap junction halls independent pathways. Overall, connexins’ coordinated activities within the gap-junction-dependent and -independent functions converge to guarantee epidermal homeostasis and promote efficient skin renewal, repair and barrier control [31].

### 3.5. Gap Junction-Dependent and -Independent Signaling Pathways in Skin

**Figure 1:**
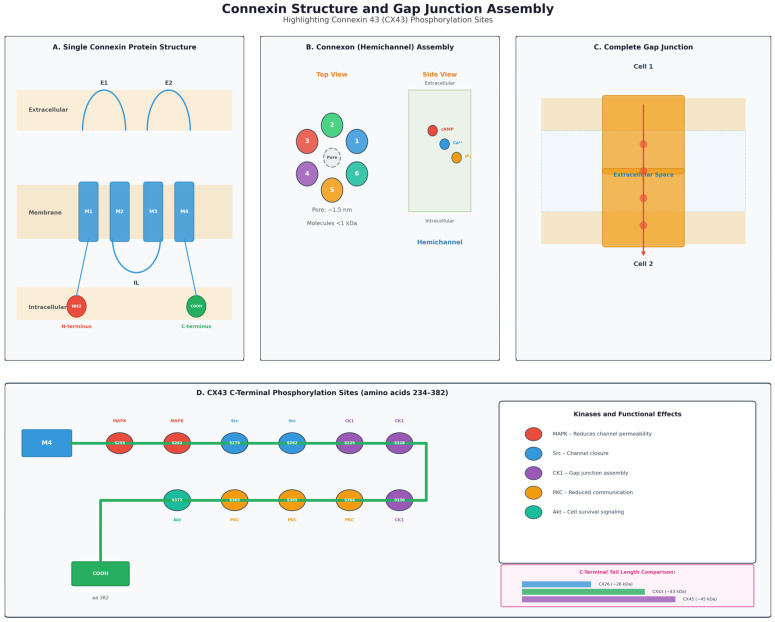
Panel (**A**) outlines a model of the connexin protein structure with four transmembrane domains, two extracellular loops that are important for docking, and a cytoplasmic N-terminal and C-terminal domain. Panel (**B**) demonstrates a model of the six connexin proteins that are necessary to assemble into a connexon (also known as a hemichannel). Panel (**C**) illustrates the two connexons docking to form a complete gap junction with the space between the connexons originally designated as the “gap” in gap junctions. Panel (**D**) shows a model of the C-Terminal tail of Cx43 with the phosphorylation sites demarcated and the kinases listed in the panel to the right.

The expression of connexins in epidermal physiology is also regulated through both gap junction-dependent and independent mechanisms. In a classical manner, connexins oligomerize into intercellular channels that directly exchange ions, second messengers, and metabolites, which allow for the synchronization of responses across the layers of keratinocytes. Beyond channel functions, connexins, or more precisely their C-terminal domain, function as signaling scaffolds that modulate fundamental intracellular pathways. The C-terminal domain of Cx43 directly interacts with signaling proteins, including Src kinases, which control the phosphorylation events responsible for the regulation of cell growth, adhesion, and cytoskeletal dynamics [32]. Furthermore, the modulation of the MAPK/ERK pathway by connexins affects the proliferation and differentiation of keratinocytes through the transduction of regulatory cues independent of direct channel communication. This ensemble of gap junction-dependent and independent functions thus integrates metabolic coordination with intracellular signal transduction to ensure proper renewal of the epidermis, wound repair, and barrier function [31]. Figure 1 outlines the structure of the connexin proteins (Panel A), the connexons (Panel B), and the complete gap junctions (Panel C). In addition, the phosphorylation sites on the C-Terminal domain of Cx43 are depicted in Panel D.

### 3.6. Connexins and Keratinocyte Motility: Implications for Tissue Dynamics

The complex roles of gap junctions and connexins are integral to maintaining homeostasis within the skin milieu and providing exciting opportunities for future melanoma treatment. Gap junctions, predominantly formed by connexin proteins like Connexin 43 (Cx43), facilitate intercellular communication between epidermal keratinocytes to coordinate cell activities and effectively regulate inflammatory signals, growth proliferation, and wound-healing processes [33]. Mutations associated with Cx43 play a role in skin signaling dysregulation affecting skin homeostasis and the development of skin disease, with evidence from models suggesting aberrant hemichannel activity plays a role in its pathogenesis. Their findings suggest that aberrant signaling pathways from gap junctions can provide insight into both skin disorders in general and potential therapies that may inhibit the malignant progression in melanoma through restoration of intercellular signaling [33]. Targeting/researching connexin-related proteins or therapeutic agents may, therefore, penetrate current melanoma therapeutic barriers to develop treatments that reinforce skin tissue integrity and compromise while limiting adverse effects.

## 4. Early-Stage Melanoma

### 4.1. Loss of Connexin Expression in Early Melanocytic Lesions and Primary Melanomas: Marked Reduction in Cx43 in Primary Lesions

Several studies, including Alaga et al. [34], demonstrate that early primary cutaneous melanomas show markedly reduced or absent connexin expression, particularly connexin 43 (Cx43), the most extensively studied connexin in skin biology. Immunofluorescence and immunohistochemical analyses of human melanoma samples indicate that Cx43 protein is almost uniformly absent in primary lesions. In rare cases where Cx43 is detectable, it remains intracellular or diffusely distributed and does not assemble into the characteristic gap junction plaques at the cell membrane that are required for functional gap junctional intercellular communication (GJIC) [34]. This pattern contrasts sharply with adjacent keratinocyte-rich epidermis, where Cx43 is abundant and organized into well-defined junctional plaques.

### 4.2. Disrupted Melanocyte–Keratinocyte Communication in Early Transformation

The loss of Cx43 in early melanocytic lesions reflects a broader breakdown in normal melanocyte–keratinocyte interactions during malignant transformation. In physiological skin, melanocytes maintain close physical and signaling contacts with surrounding keratinocytes through a combination of adhesion molecules and junctional complexes that help regulate proliferation, differentiation, and responses to environmental stress [35]. As melanocytic lesions progress toward malignancy, this coordinated communication becomes progressively impaired. Experimental work in human-derived melanoma cell lines shows that Cx43 expression is markedly decreased compared with nonmalignant melanocytic cells, supporting the concept that connexin downregulation occurs early and is linked to the transition from tightly regulated growth to more autonomous proliferation [35].

### 4.3. Systemic Evidence from Circulating and Exosomal Cx43

Clinical biomarker data supports a similar relationship between reduced Cx43 and more advanced disease. In a study of plasma-derived exosomes from 112 patients with melanoma and 50 healthy controls, overall levels of exosomal Cx43 were significantly lower in melanoma patients than in healthy individuals [36]. Within the melanoma cohort, patients with larger tumors, deeper invasion (Clark IV–V), or lymph node metastases had significantly lower circulating exosomal Cx43, whereas those with smaller primary tumors, early Clark levels (I–III), and no nodal involvement exhibited higher exosomal Cx43 levels [36]. These findings indicate that systemic Cx43 declines as tumor burden increases, and invasion progresses.

### 4.4. Implications for Early-Stage Melanoma Biology

Together, tissue-based and circulating biomarker studies support the view that early-stage melanoma is characterized by decreased or absent Cx43 expression and loss of functional GJIC within the tumor compartment [35,36]. The absence of effective Cx43-mediated communication likely removes key growth-regulatory inputs from the surrounding epidermal microenvironment, facilitating early tumor cell proliferation and local expansion. The observation that circulating exosomal Cx43 is relatively higher in patients with limited, earlier-stage disease but falls with increasing tumor thickness and nodal spread further suggests that ongoing reductions in connexin-mediated signaling accompany and may contribute to the emergence of a more aggressive melanoma phenotype [36]. Table 3 highlights the stepwise alterations in Cx43 expression to become tumor suppressors as melanocytes develop into early-stage melanoma.

### 4.5. Connexin 43 Acts as a Tumor Suppressor in Melanoma Cells: Experimental Evidence from Melanoma Cell Lines

Work in melanoma models consistently shows that connexin 43 (Cx43) functions as a suppressor of tumor development rather than a promoter of growth. When normal melanocytes are compared with melanoma cell lines, a clear pattern emerges as melanoma cells express much less Cx43 than healthy skin melanocytes. In one study using human epidermal melanocytes (HEMn) and malignant lines such as A375 and A2058, Cx43 protein levels were markedly reduced in cancer cells. When investigators forced melanoma cells to overexpress Cx43, the cells slowed their growth, formed fewer colonies, and showed reduced rates of cell division. Conversely, suppressing or knocking down Cx43 expression had the opposite effect, allowing melanoma cells to proliferate more rapidly [35]. This behavior aligns well with the idea that Cx43 participates in controlling the cell cycle and helps maintain a less aggressive phenotype. Loss of Cx43 weakens this control, giving melanoma cells greater proliferative capacity and independence from normal growth-regulating influences.

### 4.6. Tumor Growth and Metastasis in In Vivo Models

A similar pattern is seen in other human melanoma systems. Tittarelli and colleagues examined several metastatic melanoma cell lines and found that their growth characteristics varied with both the level of Cx43 expression and the degree of gap junctional intercellular communication (GJIC) [37]. When Cx43 channel activity was pharmacologically blocked, melanoma cells increased their proliferation rate. In contrast, lines that naturally expressed higher Cx43 and displayed stronger GJIC tended to grow more slowly.

These findings were reinforced in mouse xenograft models. Tumors engineered to maintain high Cx43 expression formed smaller masses and produced fewer lung metastases than control tumors derived from Cx43-poor cells [37]. The authors attributed these effects to enhanced cell–cell coupling, reduced proliferation, and an increased tendency toward apoptosis in Cx43-expressing tumors. Even though these experiments were performed with cells originally derived from metastatic lesions, the direction of the effect is consistent: higher Cx43 generally correlates with slower melanoma growth and more cell death, a relationship that is likely to be relevant earlier in the disease process as well.

### 4.7. Post-Transcriptional Regulation of Cx43 by miR-106a

There is also mechanistic support for why melanoma cells lose Cx43. Wang et al. demonstrated that microRNA-106a (miR-106a) directly targets the 3′ untranslated region of Cx43 mRNA, leading to reduced Cx43 protein production [36]. Experimental overexpression of miR-106a in melanoma cells caused a drop in Cx43 levels and a corresponding increase in proliferation. When miR-106a was inhibited, Cx43 expression was restored and cell growth slowed [36]. This provides a plausible post-transcriptional mechanism by which early melanoma lesions could downregulate Cx43, weaken gap-junction communication, and escape normal growth-regulating signals.

### 4.8. Cx43 as a Tumor Suppressor in Early Melanoma

Taken together, data from these different melanoma models are highly consistent: loss of Cx43 facilitates melanoma cell growth, whereas restoring Cx43 slows proliferation and promotes apoptosis [38,39]. Given that early melanoma tissues already show low or absent Cx43 expression, these findings support the broader concept that Cx43 functions as a tumor suppressor in early melanoma genesis, and that downregulation of Cx43 is an important step in the progression from benign melanocytic lesions to overt melanoma.

### 4.9. Circulating and Exosomal Connexin 43 as Biomarkers of Melanoma Stage: Systemic Cx43 Levels and Disease Burden

Beyond tumor tissue, emerging evidence suggests that circulating Cx43, particularly when packaged in exosomes, may serve as a biomarker of melanoma stage and disease burden. In a clinical study that analyzed plasma exosomes from 112 melanoma patients and 50 healthy controls, melanoma patients had significantly lower exosomal Cx43 overall than healthy individuals. Within the melanoma cohort, markers of earlier-stage disease, such as smaller primary tumors, lower Clark levels (I–III), and absence of regional lymph node involvement, were associated with higher exosomal Cx43 expression [36]. These observations imply that aspects of connexin-mediated communication can be detected systemically, and that circulating Cx43 may reflect underlying tumor biology.

### 4.10. Prognostic Implications of Exosomal Cx43

The prognostic value of circulating Cx43 appears to be substantial. Receiver operating characteristic (ROC) analyses in the same cohort showed good discriminatory performance, with area under the curve (AUC) values of approximately 0.77–0.78 for predicting adverse clinical outcomes [40]. Patients with higher exosomal Cx43 levels experienced significantly better five-year overall survival and disease-free survival than those with lower levels. Because exosomal cargo often mirrors active cellular processes, declining circulating Cx43 likely indicates a progressive reduction in connexin expression and signaling within melanoma cells as they acquire more invasive properties. This systemic pattern is consistent with tissue-level observations of absent or severely reduced Cx43 in primary cutaneous melanoma [34].

### 4.11. Systemic Cx43 Loss and Tumor–Microenvironment Communication

These findings also suggest that circulating Cx43 provides information about tumor–microenvironment communication [34] that may not be apparent from static tissue biopsies alone. Systemic declines in connexin levels could reflect broader disruptions in regulatory pathways that normally help restrain early tumor growth, including intercellular signaling, inflammatory responses, and cellular stress pathways. Moreover, the association between higher exosomal Cx43 and smaller, earlier-stage tumors supports the idea that loss of Cx43 is not only a marker of disease stage but may actively contribute to the transition from a confined lesion to a more invasive phenotype. Overall, available data indicate that circulating and exosomal Cx43 function as dynamic biomarkers of less advanced melanoma, reinforcing the tumor-suppressive role of connexins in the initial phases of disease. Higher exosomal Cx43 is characteristic of patients with limited, earlier-stage melanoma, whereas progressive declines in exosomal Cx43 track with increasing tumor burden and invasiveness.

### 4.12. Circulating and Exosomal Connexin 43 as Indicators of Early-Stage or Less Advanced Melanoma: Exosomal Cx43 as a Systemic Marker of Melanoma Burden

Recent evidence suggests that circulating connexin 43 (Cx43), particularly when packaged in plasma-derived exosomes, may serve as a biomarker of melanoma stage and overall disease burden [34]. In a clinical study involving 112 melanoma patients and 50 healthy controls, Shen et al. [36] found that exosomal Cx43 levels were significantly lower in melanoma patients than in healthy individuals. Within the melanoma cohort, patients with smaller primary tumors, early Clark levels (I–III), and no regional lymph node involvement exhibited higher circulating exosomal Cx43, whereas more advanced cases demonstrated substantially reduced levels. These findings indicate that alterations in connexin-mediated communication are detectable systemically and reflect biological changes occurring early in melanoma development [34].

### 4.13. Prognostic Significance of Exosomal Cx43

Circulating Cx43 also appears to have a meaningful prognostic value. Receiver operating characteristic (ROC) analyses performed by Shen et al. [34] showed that exosomal Cx43 predicted adverse clinical outcomes with good accuracy, yielding AUC values of approximately 0.77–0.78. Patients with higher circulating Cx43 levels demonstrated markedly better five-year overall survival and disease-free survival than those with lower levels. Because exosomal cargo often mirrors active intracellular processes, declining exosomal Cx43 likely reflects progressive reductions in connexin expression and signaling within melanoma cells as they acquire more invasive characteristics. This pattern parallels tissue-level observations showing that primary cutaneous melanomas frequently lack membrane-localized or functional Cx43 [34].

### 4.14. Implications for Tumor–Microenvironment Communication

These systemic findings suggest that circulating Cx43 captures aspects of tumor–microenvironment communication that may not be evident from static tissue biopsies. Connexins help regulate intercellular signaling, inflammatory responses, and cellular stress pathways; therefore, declining systemic levels may signal broader disruptions in regulatory networks that typically restrain early tumor growth. The association between higher exosomal Cx43 and smaller, early-stage tumors further supports the idea that the progressive loss of connexin expression contributes to the transition from a localized lesion to a more invasive phenotype.

### 4.15. Overall Interpretation

Together, these findings support a model in which circulating and exosomal Cx43 function as dynamic biomarkers of early-stage or less advanced melanoma [34,35,36]. Higher exosomal Cx43 is characteristic of limited, early-stage disease, while progressive reductions in circulating Cx43 track with increasing tumor thickness, nodal involvement, and overall aggressiveness. Although tissue Cx43 is already diminished in early lesions, the continued systemic decline in exosomal Cx43 appears to reflect the escalating breakdown of connexin-dependent communication as melanoma progresses [34,36]. The following section will explore how these patterns evolve further in metastatic disease, particularly in relation to the mislocalization and aberrant re-expression of Cx43 in advanced melanoma.

## 5. Late-Stage Melanoma

### 5.1. Connexin Dysregulation and Functional Re-Emergence as Instigators of Metastatic Progression

The progression of melanoma from a radial-growth primary lesion to a systemic metastatic malignancy involves a coordinated rewiring of cell–cell communication pathways, many of which are mediated by connexin proteins. Connexins were historically characterized as tumor suppressors due to reduced gap junctional intercellular communication (GJIC) in early melanoma, but contemporary human studies demonstrate that advanced melanoma selectively re-expresses or upregulates specific connexins, most notably Connexin 26 (Cx26) and Connexin 43 (Cx43), to facilitate metastasis [38,41]. This represents a stage-dependent phenotypic shift rather than a simple loss-of-function model. The current body of human melanoma literature now shows that connexins are active drivers of metastatic dissemination, endothelial engagement, and distant organ colonization [39,42].

### 5.2. Connexin-Mediated Melanoma–Endothelial Coupling as a Prerequisite for Metastasis

Ito et al. first established that metastatic melanoma cells form functional heterocellular gap junctions with vascular endothelium through Cx26 and Cx43, which enhance intravasation via calcium flux synchrony, cytoskeletal remodeling, as well as increased endothelial adhesion [43]. Inhibition of gap junction formation significantly reduced metastatic deposition in ex vivo human vein models and in vivo assays and showed that connexins are not bystanders but active components of metastatic machinery.

Saito-Katsuragi et al. extended these findings by demonstrating that Cx26 is elevated in human metastatic melanoma lesions, and that Cx26 expression increases endothelial adhesion and metastatic competence. Suppression of Cx26, however, reduces diapedesis and metastatic burden [44]. Collectively, these studies confirm Cx26 as a pro-metastatic connexin, selectively enriched in late-stage melanoma.

### 5.3. Extracellular and Circulating Connexins as Indicators of Advanced Disease Biology

**Figure 2:**
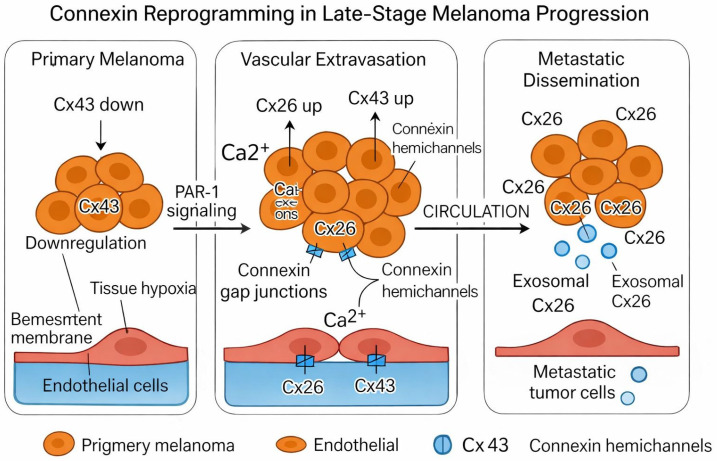
Connexin reprogramming in late-stage melanoma. Stage-dependent shifts in Cx26, Cx43, and Cx46 support metastatic progression through endothelial adhesion, gap junction formation, Cx43 membrane relocalization, and hypoxia survival. Exosomal Cx43 reflects systemic disease behavior.

Circulating exosomal Cx43 also reflects the behavior of systemic disease. Shen et al. [36] identified plasma-derived exosomal Cx43 as a clinically relevant biomarker in melanoma patients and showed that reduced circulating Cx43 correlates with higher TNM stage, recurrence, and diminished survival [36]. Although circulating Cx43 decreases while tissue Cx43 increases, this still supports the idea that altered connexin levels reflect biologically aggressive melanoma, whether inside the tumor or in circulation. Figure 2 illustrates a model of how Cx26 and Cx43 are upregulated in late-stage melanoma progression and how that leads to vascular extravasation and metastasis.

### 5.4. Regulatory Networks Controlling Connexin Expression in Late-Stage Melanoma

Connexin expression is shaped by both transcriptional and post-transcriptional networks. Beyond PAR-1–mediated activation of Cx43, Wang et al. showcased how miR-106a directly suppresses Cx43, which enhanced proliferation and reduced gap junction intercellular communication (GJIC) [35]. This illustrates competition between progression-associated signaling and oncogenic microRNAs that tune connexin levels across melanoma stages.

Beyond Cx26 and Cx43, Connexin 46 (Cx46) has emerged as an additional determinant of late-stage aggressiveness. Cx46 is highly expressed in malignant melanoma and enhances survival under hypoxic conditions characteristic of advanced disease, which promotes invasion and resistance to apoptosis [45]. This adds a third connexin to the late-stage metastatic profile and highlights the scope of connexin reprogramming in advanced melanoma.

At the same time, advanced melanoma demonstrates a shift in Cx43 localization from intracellular stores back to the plasma membrane, a redistribution required for functional gap junction formation and strongly associated with metastatic competency in specimens of human tissue [43].

### 5.5. Therapeutic Targeting Implications for Late-Stage Melanoma

The stage-specific dependence of metastatic melanoma on connexin-mediated communication suggests several therapeutic entry points. PAR-1 signaling represents a particularly promising target: inhibiting or silencing PAR-1 markedly reduces Cx43 expression, endothelial adhesion, and metastatic dissemination, while restoring Cx43 reverses these effects, establishing this pathway as a therapeutically actionable mechanism [39].

Modulation of connexin hemichannel activity or suppression of heteromeric gap junction formation may impair melanoma–endothelial coupling required for vascular extravasation. Targeting connexin-regulated calcium flux or second-messenger transfer may disrupt metastatic signaling circuits that depend on GJIC. Connexins may ultimately serve as adjuvant molecular targets that are positioned alongside immunotherapy, anti-invasive agents, or microenvironment-directed therapies [42,44].

### 5.6. Synthesis and Clinical Implications

Human tissue analyses, mechanistic cell-based assays, and in vivo metastasis models collectively demonstrate that Cx26, Cx43, and Cx46 are actively repurposed by late-stage melanoma to support metastatic dissemination [36,39,44]. These connexins enhance endothelial adhesion, vascular invasion, organ-specific colonization, and survival within hostile microenvironments, while also serving as potential biomarkers of aggressive disease. Rather than passive markers or uniform tumor suppressors, connexins exhibit stage-dependent functional plasticity, with re-expression enabling metastatic progression. These insights position connexins as emerging therapeutic targets, prognostic indicators, and mechanistic cornerstones of advanced melanoma biology.

### 5.7. Context-Dependent Re-Expression of Connexin 43 in Advanced Melanoma

Cx43 plays a more complex role than Cx26. While isolated overexpression of Cx43 in melanoma cell lines decreases proliferation and colony formation, this effect does not represent physiological metastatic behavior. Instead, late-stage melanoma re-expresses and transcriptionally upregulates Cx43 through progression-associated signaling pathways [39].

Villares et al. explained that metastatic melanoma overexpresses the thrombin receptor PAR-1, which helps to drive transcriptional activation of Cx43 through AP-1 and SP-1. PAR-1 silencing markedly reduced Cx43 levels, invasion, endothelial adhesion, and metastatic spread. Restoration of Cx43 in PAR-1-silenced cells rescued the metastatic phenotype which confirmed Cx43 as a required downstream effector of PAR-1–driven melanoma progression [39].

In late-stage melanoma, Cx43 is not only produced in higher amounts, but also moved back to the cell surface, where it can form functional gap junctions. This return of Cx43 to the membrane is strongly linked to the tumor’s ability to metastasize in human samples [43].

### 5.8. Connexins as Determinants of Brain Metastatic Colonization

Brain metastasis is a hallmark of advanced melanoma and connexins are key mediators of this process. Stoletov et al. demonstrated that Cx43 and Cx26 are essential for brain endothelial adhesion, vascular co-option, and early metastatic lesion stabilization in vivo [42]. Connexin knockdown impaired melanoma cell attachment to brain endothelium and reduced intracerebral metastatic formation, identifying connexins as prerequisites for melanoma neurotropism.

### 5.9. Human Clinical Evidence for Connexin Re-Emergence in Metastatic Melanoma

Human metastatic tissue analyses reinforce these mechanistic findings. Both Cx26 and Cx43 are significantly upregulated in metastatic human melanoma specimens compared with primary lesions, with Cx26 expression correlating with lymph node involvement and distant spread [44]. Cx43 shows stage-dependent redistribution from cytoplasmic compartments to the plasma membrane in human metastatic nodules in a similar way, which indicates restored functional gap junction signaling during metastatic progression [41]. These data confirm that connexin reactivation is a defining biological feature of human metastatic melanoma. The following section reviews the current therapeutic treatment for melanoma, outlining the advantages, limitations, and a potential role for gap junction regulation in therapeutic treatment.

## 6. Therapeutic Interventions—Clinical Relevance and Limitations

Melanoma is managed with a variety of different therapeutic strategies, each developed to address a different vulnerability of the disease and each accompanied by advantages and important limitations [1]. Although progress across the field has been substantial, no single modality has proven universally effective, and the clinical outcome often depends on decision-making that balances efficacy, toxicity, accessibility, cost, and quality of life. In practice, patients encounter a therapeutic landscape defined not by one “best” option, but by a series of trade-offs that must be considered in the context of disease stage, tumor biology, and individual priorities. It is therefore essential to evaluate existing treatments with a critical perspective, acknowledging both their contributions to patient survival and the challenges that motivate continued innovation [1]. The principal therapeutic approaches currently used in melanoma are summarized below, as well as the strengths and shortcomings that define their role in modern clinical care. The relevance of connexins and gap junctions in each treatment modality is also described.

### 6.1. Immunotherapy

In contemporary melanoma practice, immune checkpoint blockade targeting PD-1, CTLA-4, and more recently LAG-3 represents the backbone of systemic therapy across respectable high-risk and metastatic disease, complemented by cellular therapies, intralesional approaches, and combination strategies for brain metastases [46]. Immunotherapy has reshaped the treatment landscape of melanoma, but tumor control still depends on effective communication between immune effector cells and the tumor microenvironment. Increasing evidence shows that connexins (particularly Cx43) and their associated gap junction channels participate directly in this dialog. These findings suggest that the success or failure of modern immunotherapy is influenced by the integrity, location, and context of connexin-mediated intercellular communication. Within this broader framework, connexins and gap junctions emerge as a factor that can modulate antigen presentation, T-cell activation, and effector killing, and may ultimately help explain heterogeneity in immunotherapy response.

Functional gap junctions between dendritic cells enable the exchange of melanoma antigens, a process that enhances cross-presentation and improves priming of tumor-specific cytotoxic T cells, reinforcing one of the foundational steps that checkpoint inhibitors attempt to amplify [47]. This antigen-sharing mechanism is further supported by the observation that gap junctions accumulate at the immunological synapse during T-cell activation, where Cx43 facilitates calcium signaling and cytokine production required for full effector function [48]. At the tumor–effector interface, Cx43-rich junctions form at cytotoxic synapses between T cells and melanoma cells and directly support granzyme-mediated apoptosis of the target cell, strengthening the idea that gap junctions can function as a physical and biochemical conduit for immune killing [49].

However, connexin signaling has a dual nature. Under hypoxic conditions (a hallmark of immune-resistant tumor niches), melanoma cells use Cx43 channels to transfer miR-192-5p to dendritic cells and cytotoxic T cells, impairing their killing capacity and enabling localized immune escape despite systemic immunotherapy [50]. Even more strikingly, forced overexpression of Cx43 in melanoma reduces tumor proliferation, migration, and metastasis in vivo, supporting a tumor-suppressive phenotype that aligns with the immune-stimulatory role of connexins seen under normoxic conditions [50]. Together, these studies point toward an emerging model in which the contribution of Cx43 to immunotherapy depends not simply on its abundance, but on the microenvironment in which melanoma cells and immune cells interact.

### 6.2. Chemotherapy

Although the modern treatment of melanoma is dominated by immunotherapy and targeted molecular inhibition, chemotherapy still occupies a meaningful clinical space. Historically, dacarbazine and temozolomide achieved modest response rates and transient disease control, typically without an overall survival benefit [51]. The chief practical strengths of chemotherapy lie not in outcome metrics but in characteristics that occasionally make it the only feasible choice: oral dosing for temozolomide, confirmed CNS penetration, predictable hematologic toxicity, and utility for symptom palliation when rapid control is needed and other modalities are unavailable or contraindicated. Simultaneously, its disadvantages are substantial: low response rates, limited duration of benefit, cumulative hematologic toxicities, and negligible impact on long-term survival. Taken together, these features define chemotherapy as a tool best reserved for clinical scenarios where more advanced interventions cannot be used.

The cytotoxic potential of chemotherapy does not act upon isolated tumor cells; rather, its success or failure is partially determined by the communication networks that melanoma cells establish with their surrounding microenvironment. Astrocyte–melanoma gap junctions in the brain provide the clearest example. In metastatic niches, astrocytes form connexin-based channels (particularly Cx43) with melanoma cells, allowing bidirectional exchange of calcium and survival signals that blunt the apoptotic effects of chemotherapeutic agents [52]. In parallel, astrocytes receiving pro-apoptotic signals from damaged melanoma cells respond by activating STAT1-dependent inflammatory programs and transferring survival cues back through the same Cx43-rich gap junctions, further shielding melanoma cells from chemotherapy-induced cell death [53]. These observations reveal chemotherapy resistance not as an intrinsic defect within melanoma cells alone, but as a gap junction–facilitated cooperative phenomenon that is spatially enriched in the brain.

There are two important takeaways here. First, chemotherapy resistance cannot be fully understood without considering the coupling between melanoma cells and stroma. Second, connexins themselves are not inherently “pro-resistance”; their function depends on context. Cx43 overexpression in melanoma outside the CNS has been shown to reduce proliferation, migration, and metastatic potential, providing evidence for a ‘tumor-suppressive’ role. This effect contrasts sharply with the role connexins are observed in playing brain metastases, wherein they have been shown to be ‘chemoprotective,’ causing melanoma cells to become more resistant to the effects of chemotherapy [54]. Thus, the effect of connexins on chemotherapy outcomes reflects local biology rather than absolute molecular identity.

### 6.3. Surgery

Surgical resection remains the defining therapeutic intervention for melanoma, not because it reflects the newest innovations, but because it is the only modality with proven curative potential in early-stage disease. Wide local excision with stage-appropriate mar-gins balances durable local control with tissue preservation, while sentinel lymph node biopsy (SLNB) has become the standard method for staging and risk stratification rather than a treatment in its own right [55]. Completion Lymph Node Dissection (CLND), once routine, is now selectively avoided due to lack of survival benefit and increased risk of lymphedema [56]. Within this framework, the principal advantages of surgery are straightforward: immediacy of tumor removal, avoidance of systemic toxicity, and prog-nostic information from tissue and nodal pathology. The disadvantages are equally clear: surgery alone is insufficient once melanoma has spread, and even in high-risk resected disease, recurrence is common enough that adjuvant systemic therapy is now standard.

The interface with connexins and gap junction biology becomes relevant when con-sidering why surgical clearance is more effective in some settings than others. Cx43-me-diated gap junctions have been implicated in limiting invasion and metastatic initiation by promoting adhesion, regulating cytoskeletal dynamics, and reducing migratory capac-ity in melanoma cells [57]. In preclinical models, forced overexpression of Cx43 diminishes primary tumor growth and sharply reduces metastatic spread, effectively shifting mela-noma toward a phenotype that is both less invasive and more amenable to complete sur-gical eradication [50]. Conversely, loss or mislocalization of Cx43 correlates with increased motility and metastatic competence, suggesting that connexin dysfunction is a molecular step that enables metastasis before the opportunity for curative surgery has passed [38]. From this perspective, the success of surgery depends not only on anatomical timing and detection, but also on the biological state of the melanoma cell–cell communication net-work at the moment of diagnosis.

### 6.4. Radiation

Although melanoma was once viewed as radioresistant, advances in radiation deliv-ery and a better understanding of how locoregional therapy interfaces with systemic im-munity have repositioned radiation as a critical tool for controlling focal disease [58]. Its modern utility arises not from broad cytotoxicity, but from precision: stereotactic radio-surgery (SRS) allows high-dose ablation of individual lesions with excellent local control, especially in the central nervous system where metastatic involvement is both common and clinically impactful. In this sense, radiation is most valuable at the intersection of anatomy and biology: a local therapy that gains relevance as systemic agents reshape pat-terns of response. The strengths of radiation lie in its ability to achieve rapid control of symptomatic or threatening lesions, relieve neurologic complications, and serve as a tar-geted adjunct when systemic therapy provides incomplete clearance. Because radiation does not influence disseminated disease, its limitations are equally clear: its benefit is spa-tially restricted, risks such as radionecrosis or neurocognitive decline must be considered, and durable survival generally depends on its integration with immunotherapy or tar-geted agents rather than its use as a standalone modality.

The interaction between radiation and connexin biology explains why radiation re-sponses can vary across microenvironments. Functional Cx43-based gap junctions permit the transmission of radiation-induced bystander signals (reactive oxygen species, calcium fluxes, and apoptotic cues) from irradiated to neighboring non-irradiated melanoma cells, effectively expanding the cytotoxicity of a highly localized intervention [49]. Experimental work demonstrates that gap junction coupling increases overall tumor cell kill following radiation exposure, converting focal irradiation into a multicellular response rather than a single-cell event [59]. Yet this process is not uniformly maintained. Hypoxic regions of melanoma downregulate or internalize Cx43 after irradiation, reducing intercellular cou-pling and creating radioresistant pockets within the same lesion [40]. Echoing the conclu-sions of the previous therapeutic modalities, these observations emphasize that radiosen-sitivity is shaped not only by the intrinsic properties of melanoma cells but also by the condition of the communication networks that link them.

### 6.5. Nanotechnology

Nanotechnology has emerged as a therapeutic platform in melanoma not because it presents a novel mechanism, but because it reframes how therapeutic molecules behave in complex tumor environments [60]. Conventional drugs face familiar obstacles: rapid clear-ance, poor solubility, limited tumor penetration, and systemic toxicity, all of which derive from the chemical constraints of the free drug. Nanocarriers, by contrast, allow the re-searcher to re-engineer the pharmacologic identity of the same molecule, dictating how it circulates, where it accumulates, and when it is released [60].

The advantages of nanotechnology in melanoma generally fall into three categories. First, nanoscale carriers improve drug accumulation in tumors through enhanced perme-ability and retention and by protecting the payload from premature degradation: nanocar-riers shield chemotherapeutics from rapid renal clearance and enzymatic breakdown, not-ing that polymeric micelles and liposomal constructs persist longer in circulation and ac-cumulate at higher concentrations within solid tumors compared with free drugs [60]. Second, they allow the safe delivery of highly potent or synergistic drug combinations that cannot be co-administered systemically due to incompatible pharmacokinetics: nanocar-riers enable co-encapsulation of agents such as doxorubicin with natural compounds, tar-geted inhibitors, or immunomodulators, ensuring synchronized delivery, controlled stoi-chiometry, and reduced off-target toxicity [61]. In melanoma models, these co-delivery systems maintain the integrity of combination regimens that would otherwise exhibit rapid dissociation, antagonistic metabolism, or prohibitive toxicity if given in their free forms. Third, newer carrier systems incorporate microenvironment-responsive triggers (pH, redox potential, protease activity, etc.) enabling localized release within the mela-noma niche rather than uniform exposure throughout the body: stimuli-responsive nano-particles remain stable in circulation but rapidly unload their payload in the acidic, en-zyme-rich melanoma microenvironment, thereby enhancing cytotoxic precision and min-imizing systemic exposure [62]. These features are attractive not because they guarantee therapeutic breakthrough, but because they allow a controlled, tunable interface between pharmacology and tumor biology.

The disadvantages of nanotechnology are also clear. The success of nanomedicine in preclinical melanoma models does not consistently translate to human tumors, where vas-cular heterogeneity, immune surveillance, and stromal density can diminish nanoparticle accumulation [61]. Manufacturing reproducibility, regulatory hurdles, and variability in patient-level physiology further complicate clinical deployment [61]. As such, nanotech-nology should be viewed not as a monolithic solution but as a modular toolkit: useful when the biology aligns, less useful when the tumor behaves in ways that restrict nano-particle access.

The effects of nanotechnology on intracellular communication are an emerging field of research. Gap junctions regulate the intercellular transfer of ions, metabolites, and small signaling molecules, but nanoparticles themselves are far too large to traverse these chan-nels. However, the cytotoxic or immunomodulatory signals they induce may propagate through Cx43-dependent networks at the tumor periphery. Although no current mela-noma nanotherapy explicitly targets connexins, the broader literature on bystander signal amplification through Cx43 provides a conceptual bridge: oxidative or stress-related sec-ondary messengers generated in nanoparticle-treated cells can diffuse to neighboring cells through functional gap junctions, extending the effective radius of action [63]. In this sense, connexins do not mediate nanoparticle delivery but may shape how nanotherapy-induced stress signals spread through melanoma cell clusters.

### 6.6. Novel Therapies

Novel strategies for melanoma increasingly emphasize disruption of the cellular and microenvironmental conditions that sustain tumor growth. Within this landscape, con-nexins and gap junctions are emerging as regulators rather than bystanders, offering new opportunities to enhance or reshape therapeutic response. Advances in engineered im-munotherapies are expanding melanoma treatment options. Personalized neoantigen vac-cines induce robust intra-tumoral T cell responses in early trials, suggesting a role for im-mune priming strategies that integrate seamlessly with checkpoint blockade [64]. This is particularly relevant to connexin biology, as Cx43 gap junctions accumulate at the immu-nological synapse and facilitate antigen transfer and cytotoxic signaling between T cells and melanoma cells [49].

Epigenetic modulators, metabolic inhibitors, and innate immune agonists such as STING pathway activators are being evaluated for their ability to reshape the inflamma-tory and metabolic conditions within melanoma [65]. In other cancer models, cGAMP transfer through gap junctions amplifies STING-mediated signaling across neighboring cells, suggesting a mechanistic rationale for exploring similar interactions in melanoma [53]. If validated, such pathways would place connexins directly at the center of novel innate immune–based therapies.

Finally, the most direct integration of gap junction biology into melanoma therapy comes from combination strategies that intentionally exploit Cx43-dependent signal prop-agation. A small-molecule combination induces melanoma apoptosis through a positive feedback loop in which Cx43 gap junctions transmit death signals to adjacent tumor cells, increasing overall therapeutic efficacy [54]. Although preclinical, this work provides clear proof-of-concept that enhancing intercellular communication can expand the radius of drug activity and may improve the depth of response in otherwise heterogeneous tumors

## 7. Conclusions

This review outlines how connexins can play diverse and even opposite roles depending on the stage of melanoma. On one hand, a loss of connexin, mediated gap junctional intercellular communication is typically seen during the initial stage of tumor formation; On the other hand, a selective reexpression of certain connexins is observed at later stages, which helps tumor cells to spread.

Cx43 suppresses tumors by enabling communication between melanocytes and keratinocytes which is growth, inhibitory stage early melanoma [35,36,37]. However, at late stages of severely disease through PAR, 1 oncogenic pathway and invasive capacities, the transcriptional reactivation of connexin 43 thus also acquiring endothelial adhesion, extravasation, and metastatic dissemination abilities. Similarly, Cx26 has been shown to be locally overexpressed in metastatic melanoma lesions, where it is able to promote endothelial coupling and brain colonization [42]. In addition, Cx46 may facilitate melanoma cell survival under the hypoxic environment of advanced tumors and thereby enhance the late, stage aggressiveness.

The controversy still exists over whether Cx26 and Cx30 are really expressed in melanoma cells themselves or not. This doubt is raised based on the fact that multiple independent research efforts employing immunofluorescence, immunohistochemistry, and qRT, PCR have consistently failed to detect these connexins in melanocytic naevi, primary melanomas, cutaneous metastases, and human melanoma cell lines [66,67,68,69]. However, murine models and database analyses have indicated that there is a positive correlation between the level of Cx26 expression and the metastatic potential of the cells [42]. Resolving these conflicting results means that a careful study has to be done using standardized detection methods over well, characterized human and murine cohorts.

However, research shows that Cx26 and Cx30 get induced in the epidermal tumor microenvironment area near malignant tumors, and their level of expression is associated with tumor thickness and metastasis [66,67]. Although, it is still not clear if this connexin remodeling is a driving force of tumor development or a passive indication that the tumor is already on its way to becoming more malignant. In addition, the exact molecular mechanisms underlying the transformation of Cx43 from a tumor suppressor to a metastasis promoter still need to be investigated in more detail. The existing data show that the change is not a contradiction but rather a dependency on the context, determined by variations in the composition of the stroma, microenvironmental factors such as hypoxia, the type of coupling partners/subcellular interaction, and the difference between experimental overexpression in isolated cell lines and physiological re, expression driven by progression, associated signaling. These observations have substantial implications for therapy.

The understanding that connexins behave differently according to the stage of disease and site of organ necessitates very specific therapeutic strategies targeting gap junctional communication. A nonselective modulation of the connexin function may even be harmful since it may promote metastatic progression rather than the suppression of tumor growth.

Considering this, the capacity to use circulating exosomal Cx43 as a clinical biomarker for melanoma staging and prognosis is a very attractive prospect, but it has hardly been studied. Prospective clinical studies will be necessary to validate the use of exosomal Cx43 in making treatment decisions and predicting patient outcomes with a high degree of confidence. Resolving these issues will play a crucial role in paving the way for the development of targeted, context, sensitive therapies based on the advances in connexin biology that will finally lead to the improvement of the survival of melanoma patients.

## Figures and Tables

**Table 1:** **Classification of the 21 connexins that are expressed in the human body.** This table outlines the dual nomenclature, which addresses the molecular weight, as well as the gene family, which is based on sequence similarity and compatibility to form heterotypic gap junctions. The specific examples are outlined below which highlight the incompatibility of the Cx43 and Cx26 proteins to form heterotypic or heteromeric gap junction channels.

Human Connexin Families: Complete Reference of All 21 Human Connexins			
Connexin Classification Table			
Family	Protein Name	Gene	Approx. MW
**α-family (GJA genes)**			
α	**Cx43**	*GJA1*	43 kDa
α	**Cx46**	*GJA3*	46 kDa
α	**Cx37**	*GJA4*	37 kDa
α	**Cx40**	*GJA5*	40 kDa
α	**Cx50**	*GJA8*	50 kDa
α	**Cx59**	*GJA9*	59 kDa
α	**Cx62**	*GJA10*	62 kDa
***β-family (GJB genes)***			
β	**Cx32**	*GJB1*	32 kDa
β	**Cx26**	*GJB2*	26 kDa
β	**Cx31**	*GJB3*	31 kDa
β	**Cx30.3**	*GJB4*	30.3 kDa
β	**Cx31.1**	*GJB5*	31.1 kDa
β	**Cx30**	*GJB6*	30 kDa
β	**Cx25**	*GJB7*	25 kDa
***γ-family (GJC genes)***			
γ	**Cx45**	*GJC1*	45 kDa
γ	**Cx47**	*GJC2*	47 kDa
γ	**Cx30.2**	*GJC3*	30.2 kDa
***δ-family (GJD genes)***			
δ	**Cx36**	*GJD2*	36 kDa
δ	**Cx31.9**	*GJD3*	31.9 kDa
δ	**Cx40.1**	*GJD4*	40.1 kDa
***ε-family (GJE genes)***			
ε	**Cx23**	*GJE1*	23 kDa
**Connexin Pairing Rules**			
Heterotypic vs. Heteromeric Classifications			
These classifications describe how connexins pair to form functional gap junctions:			
Heteromeric: Different connexin proteins combine within the same connexon (hemichannel).			
Heterotypic: Two connexons composed of different connexins dock together to form a complete gap junction channel.			
Family-Specific Docking Rules			
α-connexins pair with α-connexins only. β-connexins dock only with β-connexins.			
Incompatibility Example			
**Cx26** (β-family) and **Cx43** (α-family) are **incompatible**—they do not dock or assemble together.			
**Summary by Family**			
α-family (GJA): 7 members—Cx43, Cx46, Cx37, Cx40, Cx50, Cx59, Cx62			
β-family (GJB): 7 members—Cx32, Cx26, Cx31, Cx30.3, Cx31.1, Cx30, Cx25			
γ-family (GJC): 3 members—Cx45, Cx47, Cx30.2 δ-family (GJD): 3 members—Cx36, Cx31.9, Cx40.1 ε-family (GJE): 1 member—Cx23			
Total: 21 Human Connexins			

**Table 2:** **Connexin Families, Tissue Expression, and Associated Diseases.** Classification of human connexins (Cx), that are expressed in the skin, by phylogenetic family (α, β, γ). This table outlines the expression pattern of the connexins throughout the body as well as the diseases associated with mutations or expression defects in specific connexins. This is important because any therapeutic intervention with connexins may affect multiple organ systems.

Family	Connexin	Gene	Tissue Expression	Associated Diseases
α	**Cx43**	*GJA1*	Heart, brain, skin, bone, lens, ovary (most widely distributed)	ODDD syndrome Arrhythmias Melanoma progression Wound healing defects
	**Cx46**	*GJA3*	Lens (fiber cells)	Congenital cataracts
	**Cx50**	*GJA8*	Lens (fiber cells)	Congenital cataracts
	**Cx37**	*GJA4*	Endothelium, ovary	Atherosclerosis Female infertility
	**Cx40**	*GJA5*	Heart, endothelium	Atrial fibrillation
	**Cx45**	*GJA7*	Heart, smooth muscle	Cardiac conduction defects
β	**Cx26**	*GJB2*	Inner ear (cochlea), skin, liver	Nonsyndromic deafness KID syndrome Vohwinkel syndrome
	**Cx30**	*GJB6*	Inner ear, skin	Deafness (DFNA3) Clouston syndrome
	**Cx31**	*GJB3*	Skin, nerves	Erythrokeratodermia Neuropathy
	**Cx32**	*GJB1*	Liver, nerves	Charcot-Marie-Tooth Neuropathy
	**Cx30.3**	*GJB4*	Skin (epidermis)	Erythrokeratodermia
γ	**Cx30.2**	*GJC3*	Skin, testis	Unknown
	**Cx47**	*GJC2*	Oligodendrocytes, CNS	Pelizaeus-Merzbacher Spastic paraplegia

Key Points: Connexin 43 (Cx43) is the most widely distributed connexin in human tissues, Cx43 acts as tumor suppressor in early melanoma (lost) but promotes metastasis in late-stage (upregulated), Cx26 and Cx30 mutations are leading causes of hereditary deafness worldwide, α, β, γ families show distinct tissue expression patterns and disease associations.

**Table 3:** Alterations in Cx43 Regulation in Early-Stage Melanoma.

Evidence	Findings	Implication for Early-Stage Melanoma	Source
Human Tissue (Primary Melanoma)	Cx43 nearly absent in early lesions; no formation of gap junction plaques; loss of GJIC.	Indicates Cx43 downregulation is an early event in tumor initiation.	[34]
Normal Skin vs. Melanoma Cells	Normal melanocytes exhibit minimal or absent Cx43, while keratinocytes are Cx43-rich. Melanoma cell lines show even further reductions or misregulation of Cx43 relative to healthy melanocytes.	Loss of coordinated melanocyte–keratinocyte communication supports transition to autonomous tumor growth.	[35]
Circulating/Exosomal Biomarkers	Higher exosomal Cx43 linked to smaller tumors and early Clark levels; reduced levels associated with deeper invasion and nodal disease.	Systemic decline in Cx43 reflects early tumor progression and increasing aggressiveness.	[36]
Overall Interpretation	Early melanoma shows reduced or absent Cx43 expression and impaired GJIC.	Loss of Cx43 removes growth-regulatory control, enabling early proliferation.	—

## Data Availability

No new data were created or analyzed in this study. Data sharing is not applicable to this article.

## Abbreviations

AJCC	American Joint Committee on Cancer
BBB	Blood–brain barrier
BRAF	B-Raf proto-oncogene, serine/threonine kinase
CAR-T	Chimeric antigen receptor T cell
CNS	Central nervous system
CTLA-4	Cytotoxic T-lymphocyte–associated protein 4
CTL	Cytotoxic T lymphocytes
Cx	Connexin
Cx26	Connexin 26
Cx43	Connexin 43
Cx46	Connexin 46
DC	Dendritic cell
ECM	Extracellular matrix
EMT	Epithelial-to-mesenchymal transition
EPR	Enhanced permeability and retention
ERK	Extracellular signal-regulated kinase
EV	Extracellular vesicle
GJ	Gap junction
GJA1	Gap junction alpha-1 protein (gene encoding Cx43)
GJIC	Gap junctional intercellular communication
HIF	Hypoxia-inducible factor
HIF-1α	Hypoxia-inducible factor 1 alpha
ICB	Immune checkpoint blockade
IFN-γ	Interferon gamma
IL	Interleukin
MAPK	Mitogen-activated protein kinase
MART-1	Melanoma antigen recognized by T cells 1
miRNA	MicroRNA
MHC	Major histocompatibility complex
MMPs	Matrix metalloproteinases
mRNA	Messenger RNA
NK	Natural killer cells
NSCLC	Non-small cell lung cancer
PD-1	Programmed cell death protein 1
PD-L1	Programmed cell death ligand 1
PTM	Post-translational modification
ROS	Reactive oxygen species
RTK	Receptor tyrosine kinase
SNAI1	Snail family transcriptional repressor 1
TAM	Tumor-associated macrophage
TIL	Tumor-infiltrating lymphocyte
TME	Tumor microenvironment
Treg	Regulatory T cell
VEGF	Vascular endothelial growth factor
VEGFA	Vascular endothelial growth factor A
